# How many animals are used for SARS‐CoV‐2 research? An overview on animal experimentation in pre‐clinical and basic research

**DOI:** 10.15252/embr.202153751

**Published:** 2021-09-07

**Authors:** Philipp Schwedhelm, Johanna Kusnick, Céline Heinl, Gilbert Schönfelder, Bettina Bert

**Affiliations:** ^1^ German Centre for the Protection of Laboratory Animals (Bf3R) German Federal Institute for Risk Assessment Berlin Germany; ^2^ Institute of Biochemistry and Biology University of Potsdam Potsdam Germany; ^3^ Corporate Member of Freie Universität Berlin, Humboldt‐Universität zu Berlin, Berlin Institute of Health Charité‐Universitätsmedizin Berlin Berlin Germany

**Keywords:** Economics, Law & Politics, Pharmacology & Drug Discovery, Science Policy & Publishing

## Abstract

Non‐technical summaries of research projects allow tracking the numbers and purpose of animal experiments related to SARS‐CoV2 research so as to provide greater transparency on animal use.

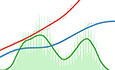

The COVID‐19 pandemic has accelerated biomedical research and drug development to an unprecedented pace. Governments worldwide released emergency funding for biomedical research that allowed scientists to focus on COVID‐19 and related drug and vaccine development. As a result, a flood of scientific articles on SARS‐CoV‐2 and COVID‐19 was published since early 2020. More importantly though, within less than 2 years, scientists in academia and industry developed vaccines against the virus from scratch: Several vaccines have now received regulatory approval and are being mass produced to immunize the human population worldwide.

This colossal success of science rests in large part on the shoulders of animals that were used in basic and pre‐clinical research and regulatory testing. Notwithstanding, animal experimentation has remained a highly controversial and heated topic between advocates for research and animal rights activists. During the past decades, European policymakers responded to the debate by enacting stricter regulations, which inevitably has increased the bureaucratic hurdles for experimentation on animals. Scientists have for long spoken out against this additional burden, arguing that both basic and translational researches to improve human health crucially relies on animal experimentation—as the COVID‐19 pandemic aptly demonstrated (Genzel *et al*, 2020).

## Transparency in animal research

The requirement of reliable data to battle COVID‐19 bolsters the argument about the need for animal experiments performed since early 2020. Yet, at the same time, it is nearly impossible to obtain even basic information on the number of animals and the purpose of the experiments, even in a leading scientific nation, such as the USA (Grimm, 2021). Other nations are more advanced in terms of transparency: For years, the European Union (EU) has been publishing annual statistics, which detail the total number of laboratory animals that were used in each member state (https://ec.europa.eu/environment/chemicals/lab_animals/reports_en.htm).

… it is nearly impossible to obtain even basic information on the number of animals and the purpose of the experiments, even in a leading scientific nation, such as the USA.

Another instrument for publishing information on animal experiments and thereby increasing transparency was introduced in 2010: Researchers in the EU are now required to summarize their planned experiments in a comprehensible way so as to inform the public about their project. The so‐called non‐technical project summaries (NTS) state the purpose and potential benefits of a research project and are submitted as part of each authorization request for an animal experiment (https://ec.europa.eu/environment/chemicals/lab_animals/nts_en.htm). The NTS also indicate the number of animals and species being used and details measures to replace, reduce, or refine animal experiments according to the 3R principle (Russell & Burch, 1959). Upon approval of the animal experiment, the NTS are published by the respective member state. In Germany, NTS are published in the openly accessible and searchable database www.animaltestinfo.de, which allows scientists working in Germany to report their *in vivo* research directly to the public (Schönfelder, 2015). Since its launch in 2015, AnimalTestInfo has grown to include more than 20 300 published NTS. In July 2021, the EU commission launched a central database to which all member states are requested to submit their NTS (https://webgate.ec.europa.eu/envdataportal/web/resources/alures/submission/nts/list). Even if these are published in their respective national languages, the database will provide a unique opportunity to get an overview of ongoing and planned research in Europe.

These summaries not only provide transparency on animal experiments for the general public but can also be used for meta‐analyses even before the results of the experiments are known and published (Bert *et al*, 2017). Here, we analyzed NTS published in Germany to detail the scope and time course of planned and approved animal experiments related to COVID‐19 research.

## Animal experiments in SARS‐COV‐2 research

We performed a text search in NTS records to identify authorized research projects related to SARS‐CoV‐2 or COVID‐19 between February 1, 2020, and July 27, 2021. Based on individual reviews of the project descriptions, we separated the NTS in two groups: projects focusing on vaccine development and others.

**Figure 1:**
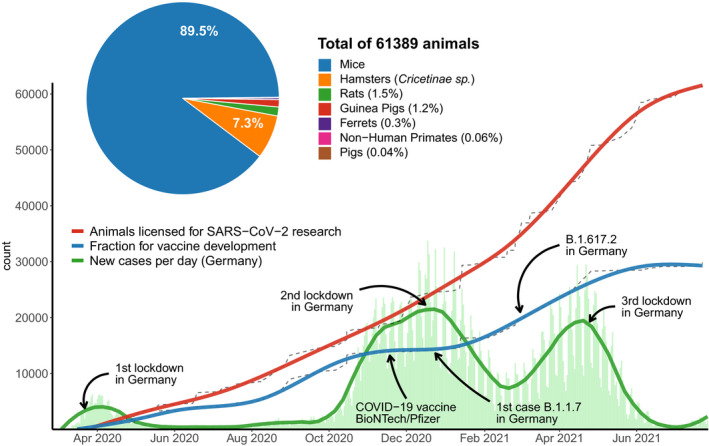
Animals used for SARS‐CoV‐2 research in Germany Between February 1, 2020 and July 27, 2021, 61,389 animals were approved for research projects related to SARS‐CoV‐2. We identified these projects by first searching for relevant keywords (SARS, COVID, CORONA) in the database www.AnimalTestInfo.de. We then manually sorted and analyzed matching NTS. The number of reported human infections in Germany started rising in early March 2020 (green curve; data from German registry for confirmed SARS‐CoV‐2 infections (Robert‐Koch‐Institute, Berlin)). The red curve represents all animals belonging to projects approved by German authorities that make a reference to SARS‐CoV‐2 research. The blue line represents all animals in projects that mentioned research or development of vaccines against SARS‐CoV‐2. Corresponding raw data are visible as grey lines. The pie chart illustrates the proportions of animal models used for SARS‐CoV‐2 research.

Research on SARS‐CoV‐2 in Germany began in‐step with the outbreak of the pandemic (Fig 1). The first related project using animals was authorized on March 16, 2020, before the first wave of infections and the first lockdown in Germany. Starting with this first project, our data show a continuous increase in research with animals on SARS‐CoV‐2. During the first 17 months of the pandemic, a total of 4,893 projects with a sum of 7,723,428 laboratory animals were authorized in Germany. Despite the exceptional situation, these numbers do not reflect an overall increase in the number of animals compared with previous years. Before the pandemic, 7.2 and 7.7 million animals were approved for research in 2018 and 2019, respectively. In 2020, this number dropped to 5.8 million and the linear projection for 2021 estimates the approval of only 4.7 million animals. The number of project approvals also decreased from about 3,500/year to 3,378 projects in 2020. Compared to the 22% decrease in the overall number of animals, the decrease in project authorizations was only 4%, though. This suggests that the decrease in animal numbers was not caused by less authorizations granted by the competent authorities.

Since March 2020, a total of 102 projects and 61,389 animals could be linked to SARS‐CoV‐2 research by means of NTS analysis (Fig 1). In other words, since the outbreak of the pandemic in Germany, only 0.8% of all animals and 2.1% of projects using animals were authorized for research on SARS‐CoV‐2. These surprisingly low numbers already include the period during the 2^nd^ and 3^rd^ wave of infections starting in Germany around October 2020 and February 2021 (Fig 1). Since, the curve of animal testing related to vaccine development has flattened, concomitant with the approval of the Pfizer/BioNTech vaccine candidate in the USA and the EU. The German‐based company CureVac also initiated the pivotal Phase 2b/3 clinical study of their vaccine candidate before the end of 2020 (Fig 1). In 2021, the approval of new animals for vaccine development has languished at a lower rate. Even after the B.1.1.7 mutation was confirmed in Germany on December 24, 2020, this was followed only by a short‐term increase in animal numbers approved for vaccine development. However, the overall number of approved animals for research on SARS‐CoV‐2 continues to rise, despite the success of various vaccine development programs. One explanation for this trend is the continued interest in disease pathology and its clinical consequences, and research on other treatment strategies against COVID‐19, such as the use of convalescent serum or monoclonal antibodies (Taylor *et al*, 2021).

… in Germany, only 0.8% of all animals and 2.1% of projects using animals were authorized for research on SARS‐CoV‐2.

Although we can only analyze animal experiments approved in Germany, it is likely that our results translate to other countries that have intensified their research on SARS‐CoV‐2. Beyond Germany, pharmaceutical companies with active vaccine development programs are based in the USA, the UK, Sweden, France, Russia, India, and others. Pre‐clinical research is often conducted in international collaborations, and thus, almost all research‐intensive countries may have ongoing involvements in SARS‐CoV‐2‐related research.

## Animal models used for SARS‐COV‐2 research

Animal experiments are invaluable to study immune functions but the predictive value of individual animal models varies widely when it comes to assessing vaccine efficacy in humans (Herati & Wherry, 2018). Looking closer at the authorized species for SARS‐CoV‐2 research in Germany, it is noticeable that primarily mice have been used as test animals (89.5%), followed by hamsters (*Cricetinae sp*.; 7.3%), rats (1.5%), guinea pigs (1.2%), ferrets (0.3%), non‐human primates (0.06%), and pigs (0.05%) (Fig 1, pie plot). Of those projects that approved the use of mice, only 22 out of 81 NTS mentioned genetically modified animals, such as humanized ACE2 mice.

This is interesting because the wild‐type mouse (*Mus musculus*) is not a suitable model to study infection with SARS‐CoV‐2. Specifically, SARS‐CoV‐2 relies on angiotensin‐converting enzyme 2 (ACE2) as a cellular surface protein to bind to its host cell. Although mice express ACE2, the mouse variant does not effectively bind the viral‐spike protein (Gurumurthy, Quadros *et al*, 2020). Therefore, diverse methodological strategies are promoted to use the mouse as a “suitable” animal model (Gurumurthy *et al*, 2020; Munoz‐Fontela, Dowling *et al*, 2020). A recent systematic review highlighted the weaknesses of the wild‐type mouse as a viral model and as a phenotype for disease as well as for studying viral and host interaction (Ehaideb *et al*, 2020).

**Figure 2:**
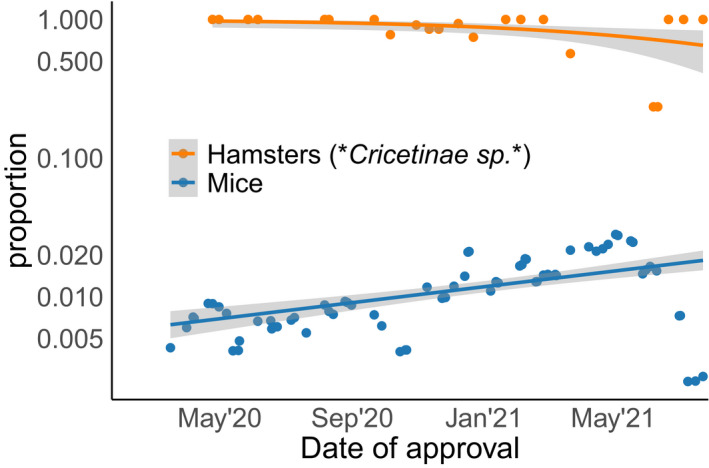
“SARS‐CoV‐2—proportion” of hamsters and mice We calculated the proportion of mice and hamsters approved for SARS‐CoV‐2 research relative to the total number of approved animals of the same species. Each data point corresponds to a project that was approved by German authorities. For each datapoint, we constructed a time‐window of 60 days around the approval date. For each time‐window, we then calculated the proportion between the sums of animals approved with and without connection to SARS‐CoV‐2 research. A value of 1 indicates that 100% of animals were allocated to projects related to SARS‐CoV‐2 research in the corresponding time‐window. We fitted these proportions separately for each species with a quasi‐binomial regression and plot 95% confidence intervals.

**Figure 3:**
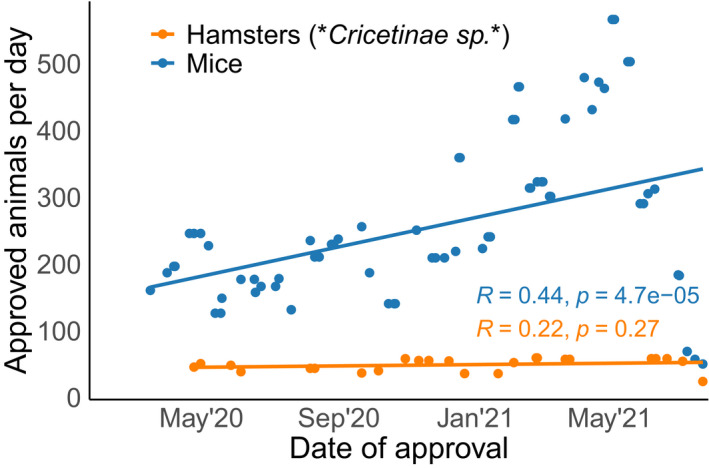
Approval rate of SARS‐CoV‐2 hamsters and mice We calculated for each approved project the approval rate in animals/day across corresponding 60‐day windows. We also calculated Pearson’s correlation coefficients for both hamsters and mice and found that the rate with which new mice were approved for SARS‐CoV‐2 research significantly increased over time.

Other research projects approved in Germany related to genetically altered viruses or testing of drug candidates on common laboratory strains. By mid‐2021, almost 2% of laboratory mice approved for use in research could be linked to SARS‐CoV‐2 projects (Fig 2). Furthermore, the rate of approval for mice in SARS‐CoV‐2 research increased significantly during the course of the pandemic (Fig 3), while this was not the case for the second most popular animal model, the hamster.

The hamster (*Cricetinae* sp.) is indeed a more suitable model, because it develops clinical features, viral kinetics, histopathological changes, and immune responses that closely mimic the disease phenotype in human COVID‐19 patients (Sia *et al*, 2020). It is especially useful for vaccine development, because passive immunization of hamsters with convalescent serum resulted in significantly reduced viral load in the respiratory tract (Ehaideb *et al*, 2020). In Germany, the first two projects using hamsters were approved by the end of April 2020. Overall, looking at the NTS data, 23.5% of SARS‐CoV‐2 project authorizations included at least some hamsters, but those represented only 7.3% of the approved animals (Fig 1). Although these numbers are very low compared with mice, the number of projects using hamsters almost tripled in comparison with the years before the pandemic. During the first six months of the pandemic, all hamsters approved for animal studies in Germany could be linked to SARS‐CoV‐2 research (Fig 2). However, this initial trend did not continue. Given the increasing number of scientific publications on the hamster model, we expected to see changes in the choice of species over time, but our data do not show such a change over the course of the pandemic.

Animal experiments are invaluable to study immune functions but the predictive value of individual animal models varies widely when it comes to assessing vaccine efficacy in humans.

A similar picture emerges for ferrets (*Mustela putorius furo*), which are an extremely valuable animal model for studying the pathogenicity and transmission of SARS‐CoV‐2. The physiology of their lungs and airways is very similar to humans, and they are popular for modeling viral respiratory diseases (Munoz‐Fontela *et al*, 2020). This is not reflected in recently authorized projects for SARS‐CoV‐2‐related research in Germany, however: only 2.9% of projects included ferrets, with a total of 185 animals during the first 17 months of the pandemic.

Rats and guinea pigs appeared in 2.7% of approved projects (Fig 1). Structural and functional modeling of SARS‐CoV‐2 entry revealed that both species are rather unsuitable to study the disease (Brooke & Prischi, 2020). Additionally, one project with 24 pigs (*Sus scrofa domesticus*) was authorized. Pigs were recently found to be not susceptible to infections with SARS‐CoV‐2 (Munoz‐Fontela *et al*, 2020).

Non‐human primates (NHP) are considered the gold standard for evaluating medical countermeasures against infections and for modeling human diseases. It is therefore surprising that, despite a lack of suitable replacement strategies to study the response in primates infected by SARS‐CoV‐2, only a small number of experiments with NHPs have been authorized to date in Germany. A single trial with six marmosets was approved during the first year of the pandemic, even though they have been found to be relatively inappropriate for SARS‐CoV‐2 research (Munoz‐Fontela *et al*, 2020). In 2021, two more projects with a total of 32 rhesus macaques were authorized, both focusing on pre‐clinical research of antibody treatments against COVID‐19, rather than development of vaccines. Since research on primates is tightly regulated in the EU, it is possible that earlier experiments were performed elsewhere to expedite critical studies. For example, BioNTech’s BNT162b‐elicited immunogenicity in rhesus macaques was investigated at the Southwest National Primate Research Centre in the USA, while all mouse studies were performed in Germany (Vogel *et al*, 2021).

## SARS‐COV‐2 research and the 3R

The surprisingly low numbers of animals approved for SARS‐CoV‐2 research might be related to the extreme pace of research. The “race” towards a vaccine in combination with the lockdown of non‐essential research led to a focus on essential studies; in this sense, the pandemic created a pressure to reduce animal numbers to the absolute minimum. At the same time, animal experiments may have been replaced with alternative methods whenever they provided faster results. On the other hand, time pressure can also create preference for well‐established research models over more suitable species. For example, scientists in Germany primarily applied for using wild‐type mice for SARS‐CoV‐2 research, although these animals were found to be not susceptible to infections. This was not an effect of the initial ramping‐up of research, because the proportion of mice that were approved for use in SARS‐CoV‐2 research continued to rise over the course of the pandemic, while the numbers of the seemingly more appropriate hamster did not (Fig 2).

An important disadvantage of animal models that develop the COVID‐19 disease phenotype is that this condition is inevitably linked to pain and suffering. Refining the experimental conditions such that no unnecessary harm is inflicted to the animals is a cornerstone of the 3R principle and plays an important role in the project authorization process for all animal experiments conducted in the EU (https://ec.europa.eu/environment/chemicals/lab_animals/related_topics_en.htm). To date, it is possible to choose from a range of animal models from humanized mice to the especially suitable Roborovski hamster (Trimpert *et al*, 2020), depending on the required severity of COVID‐19 disease progression. In this way, the number of animals experiencing severe conditions can be reduced to the absolute minimum. Ultimately, reducing suffering is what could explain the unexpected low number of “suitable” animal models. For example, critical data on the immunogenicity of vaccine candidates may be obtained from wild‐type mice without the need of a COVID‐19 disease phenotype (Rauch *et al*, 2021; Vogel *et al*, 2021).

Each NTS stands for a planned and approved research project, but its publication does not necessarily mean that the experiments will be conducted exactly as described. Rather, NTS describe the upper boundary in terms of animal numbers and severity of planned procedures. This constraint may be one explanation for the rapidly decreasing number of newly approved research animals in Germany. Project applications now include more realistic estimates of statistically required animal numbers, such that less animals are approved while the number of projects has stayed virtually constant. Ultimately, this effect should be uncovered in late 2022 by the yearly animal usage statistics that retrospectively collects the actual use of animals during the pandemic.

## Animal research benefits from transparency

Our analysis of NTS demonstrates that current trends in biomedical research can be delineated in terms of prospective animal usage. It gives a more timely and complete picture of research than publications or preprints on the same subject. However, NTS are also limited in regard to the information they contain and the provision to publish NTS does not extend to all animal experiments in research; for example, regulatory testing or organs/tissue harvesting do usually not necessitate publication of an NTS. Thus, the analysis of NTS provides a good overview of ongoing and planned research using animals, but also leaves open questions for future analyses.

The recent SARS‐CoV‐2 pandemic provides an opportunity to replace disinformation and allow for a constructive debate on the importance of animals in research.

It remains, for example, unclear why scientists preferred wild‐type mice over more appropriate animal models. Additional information, for instance about the study design, hypothesis, methods, and characteristics of the animals, would be helpful. Here, pre‐registration, that is, the publicly available registration of a full study protocol before the experiments are conducted, can bridge the gap from prospective to retrospective evaluation of a project (Bert *et al*, 2017). Pre‐registration also supports publication of all results regardless whether the study yields positive or null results. Especially in the current SARS‐CoV‐2 pandemic and given the ongoing interest in pre‐clinical research using animals, every researcher should be aware of the responsibility to inform transparently about animal experiments not only to the public but also to the scientific community.

The rapid development and approval of vaccines against SARS‐CoV‐2 is a shining example for the great potential of science. It demonstrates that public resources in research are well invested and that public trust in science is justified. Yet, in the context of the sensible topic of animal experimentation, it is important to maintain a high level of transparency to counteract misconceptions and misinformation about *in vivo* research. The recent SARS‐CoV‐2 pandemic provides an opportunity to replace disinformation and allow for a constructive debate on the importance of animals in research.

Our data demonstrate that improving transparency through NTS is a first step in this direction. Originally designed to inform the general public about animal experiments, NTS benefit also the scientific community as they enable them to obtain an overview of planned and ongoing projects in their field. This highlights once again that transparency about animal experiments ultimately serves the scientific community itself, and enables constructive debates with non‐scientists.
